# Selenium Anticancer Properties and Impact on Cellular Redox Status

**DOI:** 10.3390/antiox9010080

**Published:** 2020-01-17

**Authors:** Lolita Kuršvietienė, Aušra Mongirdienė, Jurga Bernatonienė, Jurgita Šulinskienė, Inga Stanevičienė

**Affiliations:** 1 Department of Biochemistry, Lithuanian University of Health Sciences, LT-50161 Kaunas, Lithuania; lolita.kursvietiene@lsmuni.lt (L.K.); ausra.mongirdiene@lsmuni.lt (A.M.); jurgita.sulinskiene@lsmuni.lt (J.Š.); 2 Department of Drug Technology and Social Pharmacy, Lithuanian University of Health Sciences, LT-50162 Kaunas, Lithuania; jurga.bernatoniene@lsmuni.lt; 3 Institute of Pharmaceutical Technologies, Lithuanian University of Health Sciences, LT-50162 Kaunas, Lithuania; 4 Institute of Neurosciences, Lithuanian University of Health Sciences, LT-50161 Kaunas, Lithuania

**Keywords:** selenium, redox, metabolism, anticancer

## Abstract

(1) Background: In this review, we provide information published in recent years on the chemical forms, main biological functions and especially on antioxidant and prooxidant activities of selenium. The main focus is put on the impact of selenoproteins on maintaining cellular redox balance and anticancerogenic function. Moreover, we summarize data on chemotherapeutic application of redox active selenium compounds. (2) Methods: In the first section, main aspects of metabolism and redox activity of selenium compounds is reviewed. The second outlines multiple biological functions, asserted when selenium is incorporated into the structure of selenoproteins. The final section focuses on anticancer activity of selenium and chemotherapeutic application of redox active selenium compounds as well. (3) Results: optimal dietary level of selenium ensures its proper antioxidant and anticancer activity. We pay special attention to antioxidant activities of selenium compounds, especially selenoproteins, and their importance in antioxidant defence. It is worth noting, that data on selenium anticancer properties is still contraversive. Moreover, selenium compounds as chemotherapeutic agents usually are used at supranutritional doses. (4) Conclusions: Selenium play a vital role for many organism systems due to its incorporation into selenoproteins structure. Selenium possesses antioxidant activity at optimal doses, while at supranutritional doses, it displays prooxidant activity. Redox active selenium compounds can be used for cancer treatment; recently special attention is put to selenium containing nanoparticles.

## 1. Introduction

Selenium (Se) is known to be an essential micronutrient implicated in many biological processes. Se is necessary for well-balanced functioning of many organs such as thyroid, brain, muscle, prostate, testis. Both organic and inorganic chemical forms are specific to naturally occurring Se. It is known that Se intake range between deficient and toxic levels is very narrow [1]. Therefore, it is important carefully to control intake of this element. It is important to note, that the recommended daily doses of Se depend on the geographical area [2]. The average daily intake of Se for European population recommended by World Health Organization is estimated at 40 μg (30–50 μg/day) and should not exceed 70 μg/day for adult individuals [3]. Daily intake of Se should be adequate in order to perform many functions in human body. Too low or too high intake of Se leads to formation of various disturbances in organism. Moderate deficit of this element increases the risk of infertility in men, nephropathy, prostate cancer, the occurrence of neurological diseases, ischemic heart disease, and endemic osteoarthropathy (Kashin-Beck disease), impair functioning of immune system and may lead to susceptibility to bacterial and viral infection [4,5,6,7,8,9]. However, in the case of over-supply, Se becomes toxic and may increase the risk of brain disorders, endocrine system disruption and cancer [10].

Above all, it should be pointed out that Se act as antioxidant protecting cells and tissues from oxidative stress, thus maintaining redox status in cells [11,12,13]. Supranutritional dietary level of Se ensures proper antioxidant and anticancer defence and as a result, normal functioning of immune and nervous systems. Se performes its antioxidant and anticancer activity being incorporated into selenoproteins structure. To date, 25 selenoproteins with various biological functions have been identified in humans including enzymes and non-enzymatic selenoproteins. Redox activity is known to be specific to thioredoxin reductases (TrxR), glutathione peroxidases, selenoprotein P (SelP), selenoprotein F (SelF), selenoprotein S (SelS), selenoprotein M (SelM). Se display anticancer properties due to regulation of the expression of redox active proteins and the modulation of intracellular redox status. Accordingly, this review is intended to present information published during the recent years on the biological importance, redox activity and anticancer properties of Se and Se compounds.

## 2. Aspects of Selenium Metabolism: Redox Activity of Se Compounds

The most part of organic and inorganic Se compounds are absorbed in the small intestine during digestion. Se organic compounds are much more bioavailable than Se inorganic compounds. In the digestion process, more than 80% of dietary Se organic compounds are absorbed (e.g., 98% for Se-methionine, and 84% for selenite was found to be absorbed after using single dose (200 μg) [14]. However, only about 10% of inorganic Se containing dietary compounds absorption occurs [3]. It should be pointed out that some elements such as sulfur, lead, arsenic, calcium, and iron are known to reduce the rate of Se absorption [15].

It is known that selenite is absorbed by simple diffusion, while selenate is absorbed by carrier-mediated diffusion [14]. Red blood cells take up selenite within several minutes, it is reduced to selenide by glutathione, and then transferred to plasma, where it is bound to albumin and carried to the liver and then transported to many tissues [16]. The liver is the main organ responsible for Se metabolism and synthesis of Se-containing proteins, i.e., selenoproteins as well.

The amount of Se in human body varies from 3–6 mg up to 13–20 mg, depending on the geographic location [17]. Relative high amount of Se is found in the liver, thyroid, pancreas, renal cortex, pituitary, and testis. Se content in kidney is 400–640 ng/mg wet weight and in liver is 221–340 ng/mg wet weight, while in brain is negligible; 90–110 ng/mg wet weight [18,19]. It also accumulates in hair and nails. However, the muscles, as they compose the main body mass, contain the highest amount of Se (about 46.9%), while the kidney contains only 4% of Se [3]. Two metabolic pools of Se are characteristic to human and animal bodies. One pool includes inorganic forms of Se and is important for selenoprotein synthesis. The other pool consists of organic Se forms—selenomethionine or other selenoaminoacids incorporated into proteins [17]. Se is excreted from organism by the kidneys (about 60%), with faeces (35%) and the rest through sweat and saliva (5%) [4]. Methylated selenosugars is the main form of urinary Se excretion [7]. Dimethylselenide (CH_3_)_2_Se excreted across the lung [14].

**Figure 1:**
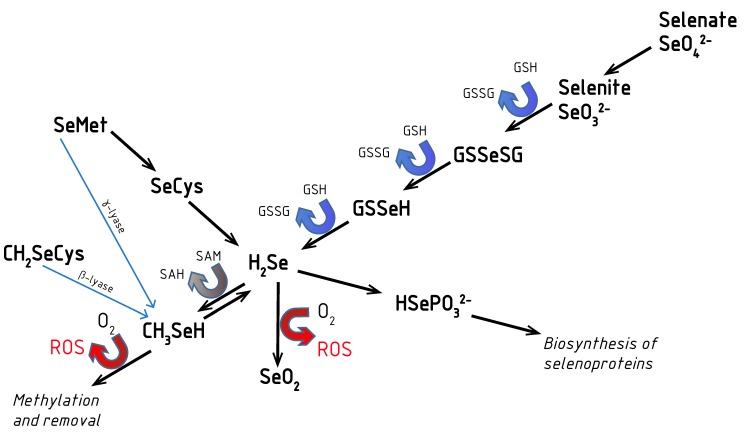
Main metabolic reactions of organic and inorganic Se compounds in humans (adapted from [20,21]). H_2_Se is formed during reduction both inorganic (selenate) and organic (SeMet), species. Both SeMet and CH_3_SeCys are enzymatically converted to CH_3_SeH. Glutathione (GSH) or other thiols are used in the reactions, when reduction of inorganic Se species occures. ROS are generated by two presented reactions. SeMet—L-selenomethionine, SeCys—selenocysteine, CH_3_SeCys—Se-methylselenocysteine, CH_3_SeH—monomethylselenol, GSSeSG—selenodiglutathione, GSSeH—selenopersulfide, H_2_Se—hydrogen selenide, GSH—reduced glutathione, GSSG—oxidized glutathione, SAM—S-adenosylmethionine, SAH—S-adenosylhomocysteine, HSePO_3_^2−^—selenophosphate, SeO_2_—Se dioxide.

Redox activity is known to be typical to both organic and inorganic Se compounds: selenite SeO_3_^2−^, Se-cysteine, Se-cystine, Se-methionine, Se-methyl-Se-cysteine, etc. As highly redox active are known hydrogene selenide HSe^−^ and monomethylselenol CH_3_Se^−^ [13]. Selenide HSe^−^ can be formed from selenite, methylselenol or selenocysteine (Figure 1). When converted into selenophosphate, selenide can be used for synthesis of selenoproteins. In Figure 1, Se metabolism including reactions when ROS (reactive oxygen species) are formed, is presented. In the selenite reaction with thiols/glutathione hydrogene, selenide is formed. The latter reacts with oxygen generating superoxide anion and elemental Se [13]. It means that Se compounds in excess act as prooxidants, generating ROS in reaction with thiols. Hence, only in proper consumption quantity when incorporated into selenoproteins structure Se compounds can externalize their beneficial redox active nature.

While the organic Se compounds have received the most attention for their antioxidant effect [22], some studies detected that inorganic Se compounds such as selenite, selenate, Se dioxide (SeO_2_), and sodium selenide (Na_2_Se) also possess similar antioxidative properties reducing the incidence of several tumor types in the experiments with animals [23,24,25,26,27,28]. Selenate in comparison with selenite is redox active and cytotoxic only when used at critically high concentration [29]. Monomethylselenol, the product of Se compounds metabolism, is considered to be most potent anticarcinogenic effect [30,31].

## 3. Selenium Status and Its Biological Importance: Selenium in the Structure of Selenoproteins

Se is necessary for many biological functions including immune defence, proper functioning of thyroid gland and reproductive system. It is considered that optimal Se serum concentrations are in ranges between 80 and 120 μg/L [32]. Risk of certain diseases is known to be increased in both cases when Se concentration is too low or too high compared with its optimal range, i.e., the relationship between Se status and risk of certain diseases is U-shaped [4]. It is supposed that serum Se level less than 80 μg/L is associated with increased risk of infertility, autoimmunity, inflammation, cancer. On the other hand, too high serum Se levels (higher than 120 μg/L) can cause hyperglycaemia, hyperlipidaemia, hyperinsulinaemia, Diabetes mellitus type 2, atherosclerosis [4]. Moreover, Se effect is known to be dependent on its concentration: at low concentration Se possesses redox active properties, while at high concentration Se exposes genotoxic and cancerogenic features [13]. Therefore, it is important to intake an adequate amount of this microelement in the diet in order to ensure proper multiple functioning in the body.

Se can perform its main and unique role when incorporated into selenoproteins. Accordingly, Se is an integral part of mammalian selenoproteins such which greatly differ in their structure, location and functions [33,34]. It is worth mentioning, that even 25 selenoproteins have been identified in humans, such as SelP, SelH, SelI, SelK, SelM, SelN, SelO, SelR, SelT, SelV, SelW, subfamilies of thioredoxin reductase (TrxR), glutathione peroxidase (GPx) and iodothyronine deiodinases (ID) [35]. Different Se species with different oxidation states (i.e., −2, 0, +2, +4, +6) can be used for the synthesis of selenoproteins in eukaryotes [3]. All selenoproteins in their structure have aminoacid selenocysteine. It is worth noting, that redox activity is specific not to all enumerated selenoproteins, but only to thioredoxin reductases, glutathione peroxidases, SelP, SelF, SelS, SelM. The location and functions of these proteins are presented in Table 1. The GPx family consists of eight members. Interestingly, not all these enzymes in their structure have selenocysteine, some of them have cysteine. In humans, following GPx members GPx1, GPx2 GPx3 GPx4, GPx6 contain Se in their structure [11]. These enzymes differ in location, but all of them catalyze reduction of hydroperoxides using reduced glutathione (GSH), which is the major low-molecular-weight thiol in cells. The main function and location of GPx6 is still unknown. Hence, GPx are well known to be an antioxidant enzyme, essential in the protection against disturbances caused by oxidative stress. It is evident that intake of Se is insufficient for full expression of GPx in many countries such as in most Europe countries and in some parts of China [36]. In mammals, TrxR family consists of three members: TrxR1, TrxR2, TrxR3 [11]. TrxR belong to the class of oxidoreductases and have NADPH as cofactor in their structure. They reduce thioredoxin, oxidized glutathione, hydrogen peroxide, lipid peroxides. It is known that TrxR family enzymes participates in different stages of carcinogenesis, from initiation to metastasis [11]. Accordingly, the maintaining of cellular redox balance takes place due to functioning of these enzymes; their expression is vital for this balance. It is surprising, that in many cancers the expression of TrxR family proteins was found to be increased [29]. Hence, inhibition of TrxR family members by particular pharmaceuticals (for example gold compounds auranofin and aurothioglucose) is one of promising strategics in cancer therapy [11].

It is well known that as constituent of selenoproteins such as cytosolic GPx, extracellular GPx, cytosolic TrxR, Se performes an antioxidant role in thyroid by eliminating free radicals formed during the thyroid hormones production [37,38]. Both GPx and TrxR performing function of antioxidant enzymes protect the thyrocytes from oxidative damage [39].

Consequently, selenoproteins are known to perform various functions such as maintaining Se homeostasis and thyroid hormone homeostasis, protection against protein and lipid damage, performing antioxidant and anti-inflammatory function, regulating cell proliferation and apoptosis [4], participating in protein folding, calcium binding and its homeostasis, eliminating misfolded proteins from endoplasmic reticulum [15]. Moreover, it is known that selenoproteins can modify and suppress tumor growth by different oncogenic pathways such as serine/threonine protein kinase, mitogen-activated protein kinase, vascular endothelial growth factor, tyrosine-protein kinase Met [11].

SeP is the main selenoprotein in plasma, composing more than 50% of total plasma Se [15,40]. This protein is known as conclusive marker of Se level in plasma reflecting supply of Se [4,6,41]. Besides, SelP is essential for Se transportation from the liver to peripheral tissues, especially to the brain and to the testicles [41]. SelP can act as chelator of heavy metals thus protecting an organism against detrimental impact of free radicals [42]. In the areas with relatively low Se concentrations SelP saturation in plasma occurs when daily Se intake is 60–70 μg for adults [35,41]. Se intake should be even greater for full expression of SelP, which main function is storage and transport of Se from liver to other organs as well [36]. There are some selenoproteins, localized to the endoplasmic reticulum, which perform regulatory function in protein folding. This group include following selenoproteins: SelF, SelM, SelN, SelK, SelT, SelS and ID2 [11,43]. For example, SelF ensures quality of disulfide bonds formation during folding process due to interplay with UDP-glucose:glycoprotein glucosyltransferase [11]. SelK and SelS were reported to be involved in degradation of misfolded proteins in endoplasmic reticulum [29]. It has been revealed recently that SelM activates the PI3K/Akt/mTOR pathway and mediate expression of matrix metallopeptidases 2 and 9 in renal cell carcinoma [44]. However, the function of some selenoproteins such as SelO, SelI is not elucidated yet [33,34].

## 4. Selenium and Cancer

It has been demonstrated by many researchers that low Se intake is associated with cancer risk [45]. However, data on the anticancer properties of Se is still contradictory. Different Se species such as inorganic, organic compounds and Se-nanoparticles possess anticancer activity, but it depends on many factors such as chemical form, dose, cancer cell type, bioavailability, stage of disease [46]. Se asserts chemopreventive activity when used at concentrations higher than optimal [29,36]. Se also is applied for cancer treatment combining with chemotherapy and radiation [47]. The utmost anticarcinogenic Se effect has been obtained when administered before or at early stage of disease development [14,40]. Se supplementation along with conventional anticancer therapies was shown to enhance the efficiency of chemotherapeutic drugs, decrease side effects and improve general condition of the patients [48,49,50]. However, more clinical trials are needed to estimate safety and efficiency of Se compounds in modulation both effectiveness and toxicity of common anticancer therapies [47]. The data obtained from already performed clinical trials are not sufficient and some of them are ambivalent.

Many in vivo and in vitro studies at supranutritional level demonstrated an anticarcinogenic effect of Se [51,52]. It has been demonstrated by Davis et al. [53] that the incidence of liver cancer was reduced by 35% in a society of 21,000 persons in China taking selenite salt supplementation. It was revealed by several studies that a 200 μg of Se per day intake, in the form of Se yeasts, reduced the risk of lung, colon, rectal, and prostate cancers [30]. Se supplementations at the same dose reduced the stomach cancer risk in subjects with low Se levels [54]. It was detected that low level of SelP is associated with higher risk of prostate, kidney, esophagus, colon, lung cancers [55]. Moreover, it has been demonstrated that Se rich plants of the genuses *Brassica* and *Allium* reduce the risk of colon cancer [30]. The pilot study by Jonklaas et al. [39] showed an association between lower blood Se levels and more advanced differentiated thyroid cancer diagnosis. In meta-analysis performed by Shen et al. [56] it was revealed that thyroid cancer correlates with decreased levels of both Se and magnesium, while increased levels of copper in comparison with control healthy group.

Se has so different anticancer effects and it is difficult to establish one predominant. Oxidative stress is known to be one of the major factors in the initiation of carcinogenesis. It has been reported that Se exhibits its anticancer effects by protection against oxidative injury [36,57,58]. Se anticancerogenic activity is related with the regulation of the expression of redox active proteins and the modulation of intracellular redox status [57]. Se is involved in the antioxidant defense system and consequently plays a relevant role in protection against oxidative stress. Many studies demonstrated that Se as an integral part of GPx increases antioxidant capacity of intracellular redox system (GSH/GSSG) and reduces cellular damage by preventing accumulation of free radical species [59,60]. GSH as an antioxidant effectively removes free radicals and other reactive oxygen species through the GPx activity, which oxidizes GSH to GSSG, and the action of NADPH-dependent glutathione reductase, which generates GSH [61]. Some types of GPxs are Se-dependent, while others are not. Hence, Se is an important element of antioxidant system that protects against metal-induced ROS. El-Demerdash demonstrated that administration of Se along with other antioxidants such as vitamin E, caused reduction of oxidative stress in the brain and other organs [62]. However, there are data about toxic dose-dependent effects of Se. The excess of Se is able to negatively affect redox status of the cell directly by oxidizing thiols, and indirectly—by generating ROS, leading to decreased redox status in body cells and thereby oxidative cellular damage [63]. Moreover, synthetic compound methyl-seleninic acid was reviewed to inhibit thiol biosynthesis due to targeting oncogene c-Myc in melanoma cells [29]. It is known that interaction of Se with metals such as cadmium, cobalt, gold, platinum, mercury can shift redox status in cells [64]. It was found recently that interaction of Se with zinc is important; metallothionein system can by negatively affected by Se due to impairment of zinc homeostasis and losing antioxidant role of this system. Such dysregulation can lead to oxidative DNA damage and cancer development [65].

**Figure 2:**
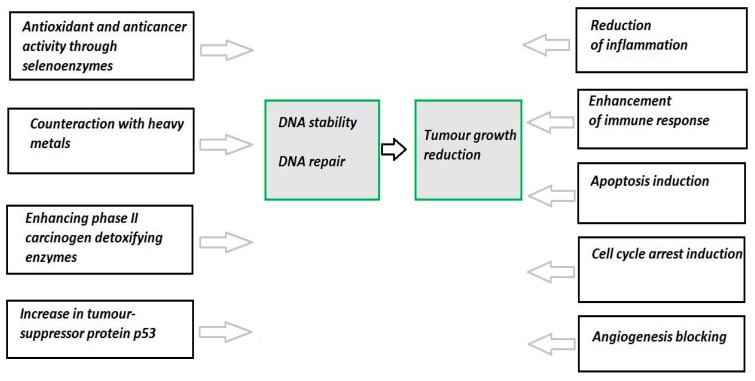
Anticancer effect of selenium may be asserted through various events and pathways in the cell. Data from studies [29,52,57,67,68,69].

The anticancerogenic effect of Se is caused not only by its antioxidative properties [66]. Different other mechanisms, identified at both molecular and genetic levels, are involved in Se anticancerogenic activity. They are represented schematically in Figure 2. These mechanisms include the ability to counteract heavy metals toxicity, maintenance of DNA stability, stimulation of DNA repair, regulation of inflammatory and immune responses, induction of cell cycle arrest and apoptosis, inhibition of local invasion and migration, blocking of angiogenesis, modulation of cell proliferation, and enhancing phase II-carcinogen-detoxifying enzymes [57,67,68]. Different Se compounds were reviewed to induce apoptosis due to different cellular effects and signaling pathways [29,46,64]. It has been noted, that supplementation with organic Se reduced various cancer metastasis in vitro and in vivo [52]. Moreover, studies in vitro and in vivo, reviewed in [68], revealed that both Se compounds and selenoproteins act as anti-metastatic agents due to inhibiting various cancer cell migration, invasion and angiogenesis.

Hence, Se asserts anticarcinogenic action depending on its chemical form, dosage and the stage of disease as well [70,71,72]. Se exerts anticancer effect through multiple mechanisms, which need to be investigated in details. Inconsistent results from various studies review the complexity of Se biology and indicate that successive research is needed to optimize benefits and reduce risk associated with Se supplementation and treatment.

## 5. Chemotherapeutic Application of Redox Active Se Compounds

Data of many both in vitro and animal studies revealed that for chemotherapeutic purposes supranutritional doses of Se are necessary. Only at higher than optimal doses Se compounds display cell growth inhibiting and cytotoxic activities [29]. It should be noted that these activities depend on Se species, dose and experimental model applied [73]. Due to high reactivity and pro-oxidative nature both metabolites selenide and monomethylselenol are effective in cancer treatment. However, there are few human trials on this issue.

Redox active Se compounds can be used for cancer treatment due to specific features of cancer cells: elevated basal ROS level, upregulated antioxidant system of protection and low tolerance to increased ROS level [3]. Se compounds assert pro-oxidative effect on neoplastic cells by some main pathways: ROS generation, oxidation of thiols in proteins and DNA binding [73,74]. As mentioned above, it is known that Se compounds can shift redox balance by oxidation of intracellular thiols, which are implicated in metabolism, transcription and signaling [29]. Abundance of proteins such as transcription factors, regulatory proteins, caspases, phosphatases, protein kinases can by modified in this way; as a consequence, their biological activity is affected and many biochemical processes e.g., calcium and iron homeostasis is impaired [29,74].

It is worth mentioning that during recent years a special attention to Se containing nanoparticles and their appliance in medicine is given [75,76,77,78,79,80]. Due to both reduced toxicity and improved targeting Se-nanoparticles are more effective in cancer treatment in comparison with other Se compounds. Diversity of nanoparticles is caused by different methods of their synthesis, i.e., chemosynthesis, biosynthesis and physical synthesis. Besides, their decoration design enables convey them to appropriate target. Poor uptake of nanoparticles by cells can be increased by changing surface charge or/and binding particular ligands on the outside surface of particles during synthesis. Supposedly, uptake of nanoparticles by malignant cells occurs via endocytosis, inside the cells nanoparticles act as prooxidants, they increased ROS formation leading to endoplasmic reticulum stress, mitochondrial membrane cleavage, apoptosis, DNA fragmentation and cell cycle arrest. Se-nanoparticles have been applied in treatment of various disturbances associated with oxidative stress and inflammation, such as cancer, diabetes, arthritis, nephropathy, liver fibrosis, drug induced toxicity [77]. Dozens of latest studies, summarized recently in the review of Tan et al. [78], demonstrated that various Se nanoparticles can be used not only for chemotherapy but for cancer diagnosis, anti-cancer drug delivery and other multiple function related to cancer as well.

## 6. Conclusions

All things considered, microelement Se is vital due to wide variety of biological functions in humans and animals. Its advantageous role in health is because of low molecular weight of Se compounds, as well as to its presence in 25 selenoproteins. It is known that thioredoxin reductases, glutathione peroxidases and some other selenoproteins such as SelP, SelF, SelS, SelM possess redox activity and regulates redox balance in cells.

In comparison with other trace elements, Se is characterized by very narrow quantitative range of concentrations between physiological status, deficiency and toxic dose. Se acts as antioxidant at optimal doses, while at supranutritional doses it displays prooxidant activity. For this reason, it is essential to precisely control intake of Se. Se supply is reflected by concentration of SelP in blood plasma. Lower intake is not sufficient for full expression of plasma SelP which is the main marker of Se status in an organism. In case of sufficient Se supply when selenoproteins are full expressed supplemental intake is not recommended.

As mentioned above, continued investigation of Se absorption, distribution and metabolism mechanisms is necessary to assess Se-related health improvement, avoiding adverse effects of Se. Different absorption and metabolism of organic and inorganic Se compounds cause different health effects of these chemical forms. Furthermore, worse bioavailability of inorganic forms determines less effective health effects compared to organic forms which are introduced directly into selenoproteins structure.

Although many studies suggest protective redox active and anticancer properties of Se, more data is needed to understand its role for therapeutic applications using synergistically with chemotherapy and radiation, especially the ability to modulate both efficiency and toxicity of anticancer therapies. Moreover, more precise evaluation is required to ascertain the interaction of Se with other metals, with other meal components and/or dietary supplements. Studies related to Se nanoparticles are considered especially promising due to typical features of nanoparticles such as low toxicity, high bioavailability and broad spectrum of appliance in biology and medicine. Se-nanoparticles are considered to be useful not only for cancer treatment, but also for drug induced toxicity and therapy, in addition to other diseases such as diabetes, bone toxicity, colitis.

## Figures and Tables

**Table 1:** Redox active selenoproteins, their location and main functions.

Selenoprotein	Location	Main Function	References
**GPx1**	cell cytosol and mitochondria	reduces peroxides to water	[11]
**GPx2**	gastrointestinal tract, liver	reduces free hydroperoxides of fatty acid and hydrogene peroxide	[12]
**GPx3**	plasma and extracellular fluid, kidneys	perform antioxidant function in plasma	[11,12]
**GPx4**	cell cytosol and cell membranes, testis	reduces lipid hydroperoxides, participates in ferroptosis (iron–based cell death)	[11,12]
**TRx1**	cytosol of liver, kidney, bone, heart cells	reduces thioredoxins	[12]
**TRx2**	mitochondria	reduces thioredoxins	[11]
**TRx3**	testis	reduces thioredoxins	[11]
**SelP**	plasma, extracellular compartment	storage and transport of Se from liver to other tissues	[12]
**SelF**	endoplasmic reticulum of liver, prostate, T-cells	possesses oxidoreductase activity, regulates protein folding	[11]
**SelS**	endoplasmic reticulum	regulates cellular redox balance	[40]
**SelM**	endoplasmic reticulum	regulates protein folding	[11]
